# Longitudinal rate of change in CSF biomarkers is associated with subsequent tau PET burden in Braak stages I and II: Findings from the BIOCARD study

**DOI:** 10.1002/alz70862_110116

**Published:** 2025-12-23

**Authors:** Nisha Rani, Abhay Moghekar, Daniel D. Callow, Kylie H. Alm, Corinne Pettigrew, Anja Soldan, Michael Miller, Jiangxia Wang, Marilyn S. S. Albert, Arnold Bakker

**Affiliations:** ^1^ Johns Hopkins University School of Medicine, Baltimore, MD USA; ^2^ Department of Neurology, Johns Hopkins University School of Medicine, Baltimore, MD USA; ^3^ Johns Hopkins University, Baltimore, MD USA; ^4^ Johns Hopkins Bloomberg School of Public Health, Baltimore, MD USA

## Abstract

**Background:**

Alzheimer's disease (AD) is characterized by the accumulation of amyloid‐beta (Aβ) and tau in the brain, years before the onset of cognitive symptoms. Understanding the association between early cerebrospinal fluid (CSF) biomarker changes and subsequent tau pathology based on positron emission tomography (PET) can provide critical insights for early detection and intervention. This study investigated whether longitudinal changes in CSF AD biomarkers were associated with tau deposition in medial temporal lobe (MTL) regions reflecting Braak stages I and II.

**Method:**

Analyses included 121 BIOCARD study participants who were cognitively unimpaired at baseline (mean age 57 years). Current tau burden was measured by ^18^F‐MK6240 PET, and prior levels and trajectories of change in CSF AD biomarkers were measured with *p*‐tau181, total tau (t‐tau), and Aβ42/Aβ40 (Lumipulse assay), collected approximately bi‐annually over a mean of 12.8 years prior to the tau PET scan. Linear mixed‐effect models examined how prior CSF biomarker levels and rates of change in these CSF markers relate to current tau PET burden, covarying for age, sex, education, APOE4 genotype, and amyloid positivity.

**Result:**

Results revealed significant associations between higher tau PET burden in Braak stages I and II and elevated prior levels of *p*‐tau181 and t‐tau, as well as lower Aβ42/Aβ40 ratios. Additionally, steeper increases in prior *p*‐tau181 and t‐tau levels and accelerated rates of Aβ42/Aβ40 decline over time were associated with greater tau burden in Braak stages I and II.

**Conclusion:**

These findings suggest that tau PET burden is preceded by early alterations and more marked changes over time in CSF AD biomarkers. This study extends our understanding of the relationship between CSF and PET measures for tracking AD disease progression.

